# Contributions of Ubiquitin and Ubiquitination to Flaviviral Antagonism of Type I IFN

**DOI:** 10.3390/v13050763

**Published:** 2021-04-27

**Authors:** Erika Hay-McCullough, Juliet Morrison

**Affiliations:** Department of Microbiology and Plant Pathology, University of California, Riverside, CA 92521, USA; erika.hay@ucr.edu

**Keywords:** interferon, flavivirus, ubiquitin, interferon antagonism

## Abstract

Flaviviruses implement a broad range of antagonism strategies against the host antiviral response. A pivotal component of the early host response is production and signaling of type I interferon (IFN-I). Ubiquitin, a prevalent cellular protein-modifying molecule, is heavily involved in the cellular regulation of this and other immune response pathways. Viruses use ubiquitin and ubiquitin machinery to antagonize various steps of these pathways through diverse mechanisms. Here, we highlight ways in which flaviviruses use or inhibit ubiquitin to antagonize the antiviral IFN-I response.

## 1. Introduction

The *Flavivirus* genus, of the *Flaviviridae* family, encompasses a group of prevalent and impactful virus species. Some flaviviruses are ancient, dating back to the 10th century in the case of dengue virus (DENV) [1], while others such as Zika virus emerged relatively recently [2]. There are upwards of 50 species of flaviviruses, but only a handful of them, such as West Nile (WNV), dengue, yellow fever (YFV), Japanese encephalitis (JEV), and tick-borne encephalitis (TBEV) viruses, afflict humans at a global scale. Depending on the flavivirus, disease symptoms range from asymptomatic to mild to severe, including vascular leakage, encephalitis, and/or shock. Flaviviruses may have multiple hosts, principally mammals and arthropods. The geographic range of arthropod vectors has expanded due to rising global temperatures and increased intercontinental travel and transport [3,4,5,6,7]. Consequently, the geographic range of flavivirus infections has broadened as well. 

Flaviviruses are enveloped, single-stranded, positive-sense RNA viruses. They enter susceptible cells through clathrin-mediated endocytosis after attachment to cell membrane receptor proteins [8]. The entire 10–11 kilobase, monopartite genome is translated by cellular ribosomes into a single polyprotein upon entry into a permissive cell. Cellular and viral proteases cleave the polyprotein into seven nonstructural proteins and three structural proteins that serve different functions throughout the viral replication cycle. The nonstructural proteins (NS1, NS2A, NS2B, NS3, NS4A, NS4B, and NS5) are involved in replication and virion assembly, while the structural proteins (capsid, C; pre-membrane, prM; envelope, E) are important for viral entry and egress from cells. To maximize the coding capacity of their tiny genomes, flaviviral proteins play multiple roles during the flavivirus life cycle. Both flaviviral structural and nonstructural proteins have roles in host immunity evasion that are distinct from their replication cycle functions. One example is the DENV protease complex NS2B3, which cleaves the viral polyprotein as well as the adaptor protein, stimulator of interferon genes (STING), thereby blocking the production of type I interferon (IFNα/β or IFN-I) and proinflammatory cytokines in response to viral infection [9,10]. Another example is NS5, an RNA-dependent RNA polymerase and methyltransferase, that also encodes IFN-I signaling antagonist functions [11,12,13,14,15,16,17,18,19,20,21,22,23,24,25,26,27,28,29].

Cellular proteins can be covalently modified by small molecules such as phosphorus, carbohydrates, ubiquitin, and methyl groups, leading to changes in protein structure, function, location, and/or stability. These post-translational protein modifications (PTMs) effectively increase protein diversity without additional gene expression. One important protein modifier is ubiquitin, a 76-amino acid molecule. Ubiquitin was discovered only 40 years ago, and determined to be essential for normal cellular protein degradation [30,31,32]. Nearly all cellular processes involve ubiquitin [33]. Extensive use of ubiquitin in cellular processes has inevitably led to viruses evolving mechanisms to hijack this molecule or control its regulators. Ubiquitin is a particularly advantageous PTM for a restricted-genome virus to utilize because the molecule is expressed in most tissues of eukaryotes, and can potentially broaden viral tropism and relieve the need to encode host-specific regulatory proteins such as E3 ligases [19,26,34]. Manipulation of ubiquitin is one strategy adapted by members of the *Flavivirus* genus [35]. In this review, we discuss the various ways flaviviruses use ubiquitin to antagonize the IFN-I response.

## 2. The Ubiquitin System

**Figure 1:**
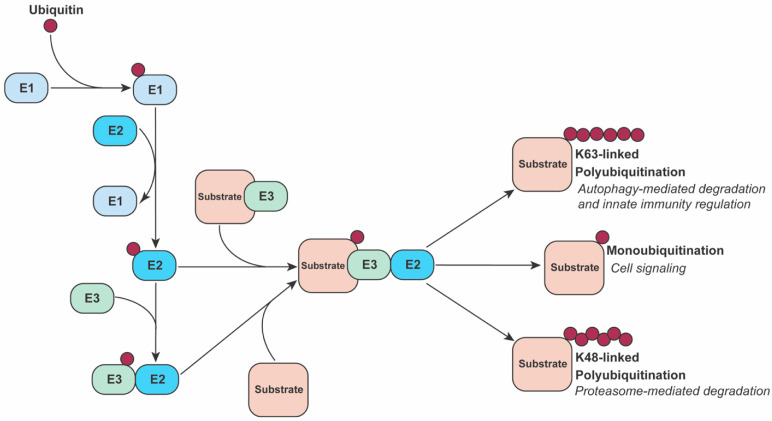
The ubiquitination process. An E1 enzyme starts the ubiquitination process by recruiting and activating a free ubiquitin molecule. This ubiquitin is then passed to an E2 enzyme through direct conjugation. After the E2 enzyme is charged with ubiquitin, multiple pathways can be taken depending on the specific E2 and E3 enzymes involved. In one common pathway, an E3 identifies both substrate and E2, then catalyzes the transfer of ubiquitin directly to the substrate. In another pathway, the E3 links the substrate and E2 enzyme, but the E2 enzyme catalyzes the transfer of ubiquitin to the substrate. Through different cascades and complexes of ubiquitinating enzymes, a substrate can start with a single ubiquitin and, through multiple rounds of ubiquitin–ubiquitin linkage conjugation, end with a chain of polyubiquitin with specific linkage types.

Ubiquitin requires a dedicated collection of enzymes to catalyze attachment to a target protein. A trio of enzymes act in concert to sequentially perform reactions necessary for ubiquitination to occur. First, an activating E1 enzyme attaches free ubiquitin to one of its thiol groups through ATP hydrolysis. Next, a conjugating E2 enzyme bonds the ubiquitin to its own thiol. Finally, an E3 ligase, which can interact with both the E2 and a specific substrate, mediates a one-step direct transfer of ubiquitin to the substrate or a two-step transfer with the E3 acting as the intermediate. There are exceptions to this categorization such as E2 enzymes acting in substrate recognition or an E3 protein acting as a scaffold while another E3 or E2 enzyme catalyzes the transfer of ubiquitin to the substrate [36,37] (Figure 1).

The functional diversity of ubiquitin regulation stems from the number of distinct enzymes encoded by the genome. Humans encode two E1s, approximately 40 E2s, and approximately 700 E3s [38]. Included in this system are negative regulators called deubiquitinases that remove ubiquitin moieties from proteins in a highly specific manner. The abundance of E3s is necessary since most are tasked with specific identification of substrates, and each E3 specializes in the ubiquitin linkage types it can catalyze. The simplest ubiquitin modification is the addition of a single ubiquitin to a lysine (K) residue of a substrate protein in a process called monoubiquitination. Multimonoubiquitination occurs when a single ubiquitin is added to multiple lysines. Polyubiquitination consists of a peptide chain of ubiquitin monomers formed through covalent bonds at one of the seven lysine residues or the N-terminal methionine residue of ubiquitin [33]. Seven ubiquitin linkage types, i.e., the K6, K11, K27, K29, K33, K48, K63, or M1 residues that link two ubiquitin molecules, direct modified proteins to their fates. 

The best studied effects of ubiquitination are targeted degradation through the ubiquitin-proteasome system (UPS). K48-linked polyubiquitination of a protein is the canonical signal for its proteasomal degradation, but can also be accompanied by other linkages such as K11-linked or K27-linked polyubiquitination [39,40,41] or heterogeneous chains [42]. Ubiquitin is also used for regulation and substrate specificity in the autophagy-lysosome pathway, which functions in bulk degradation of cellular components including proteins. The best understood linkage type associated with autophagy is K63-linked ubiquitination [43].

Not all ubiquitin linkage configurations designate a protein for destruction; there is extensive use of ubiquitination for signaling and regulation in innate immune pathways [44]. For example, K11 linkages are involved in the negative regulation of STING [45] and the interaction of mitochondrial antiviral signaling (MAVS) with retinoic acid-inducible gene I (RIG-I) [46] in the IFN-I production pathway. M1-linked linear chains are involved in inhibition of MAVS signaling and upregulation of NF-κB signaling [47,48]. K33 linkages have been reported in STAT1 transcription suppression [49] and IFN-I signaling inhibition [50]. K27 linkages are involved in downregulation [51,52,53,54] and upregulation [55,56,57] of IFN-I and proinflammatory cytokine production. No one ubiquitin linkage signal consistently activates or inhibits the protein it modifies. Instead, its fate depends on complex interactions with other cellular factors.

## 3. The Type I Interferon Response

Interferons (IFNs) initiate the innate immune response to viral infection at the cellular level [58,59,60]. The human genome encodes three classes of IFNs, type I, II and III (IFN-I, IFN-II and IFN-III), but only IFN-I and IFN-III are expressed in direct response to viral challenge [61,62,63,64]. Within the human genome, the IFN-II group includes only IFN-γ, the IFN-III group includes IFN-λ (four subtype variants), and the IFN-I group includes multiple types [65,66]. IFN-α (fourteen subtype variants) and IFN-β (one subtype variant) are the most central IFN-I molecules for activating an antiviral state.

**Figure 2:**
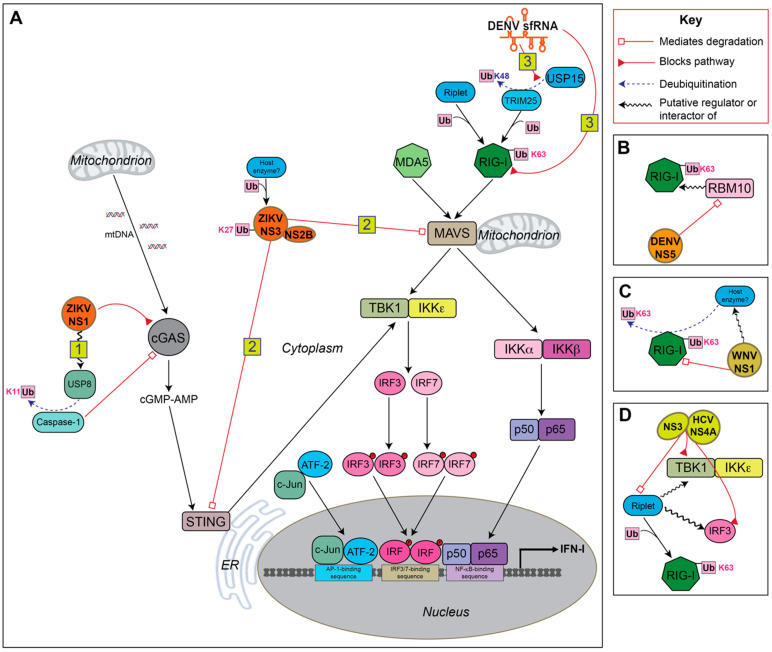
Summary of flavivirus ubiquitin-mediated antagonism of IFN production. (**A**) (1) ZIKV nonstructural protein 1 (NS1) recruits ubiquitin-specific protease 8 (USP8) to cleave ubiquitin from caspase-1, stabilizing caspase-1 and allowing increased cleavage and degradation of the pattern recognition receptor, cGAS [77]. (2) ZIKV NS3 protein is ubiquitinated by an unknown host enzyme, leading to the enhanced formation of the serine protease complex, NS32B. This leads to increased binding of STING and possibly increased STING cleavage [78,79]. NS3 also mediates ubiquitination and degradation of MAVS [79]. (3) DENV2 subgenomic flavivirus RNA (sfRNA) binds the E3 ubiquitin ligase TRIM25 and prevents its deubiquitination by USP15. This downregulates TRIM25 and results in diminished RIG-I ubiquitination and attenuated RIG-I-mediated signaling [80]. (**B**) RBM10 is a putative activator and mediator of RIG-I ubiquitination. DENV NS5 binds and mediates the proteasomal degradation of RBM10 [81]. (**C**) The interaction of WNV NS1 with RIG-I correlates with a global decrease in K63-linked ubiquitination of RIG-I. This change results in its degradation suggesting that NS1 is mediating this effect. The ubiquitin-modifying enzyme is unknown [82]. (**D**) The serine protease complex NS3-NS4A of flavivirus-related hepatitis C virus (HCV) cleaves Riplet, an E3 ligase required for ample activation of RIG-I by ubiquitination, as well as MAVS to antagonize the RIG-I/MAVS signaling pathway [83]. Riplet may also function as an E3 ligase to activate TBK1 or IRF3 directly or indirectly, an effect that is disrupted by NS4A [84].

Viral genome replication produces double-stranded RNA intermediates, which act as pathogen-associated molecular patterns (PAMPs). Cytosolic pattern recognition receptors (PRRs), RIG-I and melanoma differentiation-associated protein 5 (MDA5) recognize these PAMPs (Figure 2). RIG-I binds short dsRNA [67] and 5′ triphosphorylated single-stranded RNA [68], while MDA5 detects longer double-stranded RNA [69]. Multisite ubiquitination of RIG-I is critical for its activation and transduction in the IFN-I production pathway since this modification controls its interactions with downstream signaling factors [70,71]. Various cellular E3 ubiquitin ligases work together to regulate RIG-I ubiquitination. One well studied ligase, tripartite motif 25 (TRIM25), induces K63-linked polyubiquitination of RIG-I, leading to its interaction with the adaptor protein mitochondrial antiviral signaling (MAVS), also known as IPS-1, VISA, and CARDIF [72,73,74,75,76]. 

Another ligase that activates RIG-I is the RING-finger protein Riplet, also known as REUL and RNF135 [85,86,87]. Riplet was thought to derepress RIG-I prior to activation by TRIM25 [83]. However, more recent evidence suggests that its activity may be dominant to TRIM25′s with regard to RIG-I activation [88]. Riplet also potentially directly or indirectly activates the factors downstream from RIG-I, interferon regulatory factor 3 (IRF3) and TANK-binding kinase 1 (TBK1) [84], and has been shown to have ubiquitin-independent functions in the RIG-I-dependent signaling pathway [89]. 

Following RIG-I/MAVS interaction, a cascade of events occurs that culminates in activation of the transcription factors NF-κB, IRF3 and interferon regulatory factor 7 (IRF7) [90]. Activation of the inhibitor of nuclear factor kappa-B kinase (IKK) complex leads to derepression of NF-κB, causing NF-κB to translocate to the nucleus where it activates the transcription of proinflammatory cytokines [91]. TBK1 and IKK-epsilon (IKKε) complex phosphorylates IRF3 or IRF7, which then dimerize and translocate to the nucleus to activate transcription of IFN-β or IFN-α, respectively. 

TBK1 can also be activated by the DNA-sensing PRR adapter protein, stimulator of interferon genes (STING) [92,93]. Cyclic GMP-AMP synthase (cGAS) is a cytosolic receptor, analogous to RIG-I, that binds DNA or RNA-DNA hybrids and signals to STING, which then recruits and activates TBK1. Flavivirus infection induces the release of mitochondrial DNA, which is detected by cGAS, thereby activating the cGAS/STING pathway [94,95] (Figure 2). 

**Figure 3:**
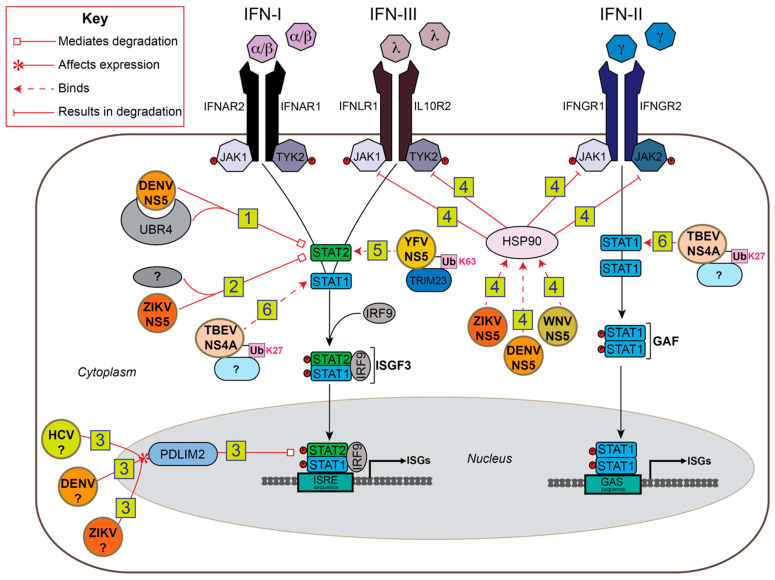
Summary of flavivirus ubiquitin-mediated antagonism of IFN signaling. (1) DENV NS5 binds both STAT2 and the E3 ligase, UBR4, to mediate proteasomal degradation of STAT2 [13,14,15]. (2) ZIKV NS5 binds an unknown ubiquitinating enzyme along with STAT2 to mediate STAT2 proteasomal degradation [17,18]. (3) Unknown proteins from HCV, DENV, and ZIKV upregulate the E3 ubiquitin ligase, PDLIM2, leading to the ubiquitination and degradation of STAT2 via the nuclear proteasome [96]. (4) NS5 of WNV, ZIKA, and JEV bind to heat shock protein 90 (HSP90) to prevent it from stabilizing the JAK proteins, JAK1, JAK2, and TYK2. Increased destabilization leads to their degradation by the proteasome [97]. (5) YFV NS5 is ubiquitinated by TRIM23 in an IFN-dependent manner, leading NS5 to bind STAT2 and inhibit its transcriptional activity [19]. (6) TBEV NS4A is ubiquitinated by an unknown ubiquitinating enzyme, and binds STAT1 to prevent its phosphorylation and dimerization; STAT2 is similarly affected but does not require ubiquitination [98].

Following the production of IFN-I and its extracellular release, IFN-I binds to the type I interferon receptor (IFNAR) (Figure 3). This results in the phosphorylation of Janus kinases, JAK1 and TYK2, which phosphorylate signal transducer and activator of transcription 1 and 2 (STAT1/2). These two proteins recruit a third protein, interferon regulatory factor 9 (IRF9), to form the interferon-stimulated gene factor 3 (ISGF3) complex. ISGF3 translocates to the nucleus and initiates transcription of interferon-stimulated genes (ISGs) by binding to the interferon-stimulated response element (ISRE) in their promoters. ISGs have diverse roles including targeted and generalized antiviral functions, as well as production of additional quantities of interferons and signal transduction proteins to amplify the response (Figure 3).

## 4. Ubiquitin-Mediated Antagonism of IFN-I Production by Flaviviruses

IFN-I is important for human protection against flavivirus infection, as demonstrated in human cell lines and mouse models of disease, as well as in human patients, where a robust and early IFN-I response is the primary influencer of viral titer and disease prognosis [99,100,101,102]. The critical importance of IFN-I in inhibiting flavivirus infection makes this pathway a prime target for flavivirus countermeasures (Figure 2).

### 4.1. sfRNA

The ubiquitination of RIG-I is critical to its activation [72, 83], and many viruses antagonize this step [103,104,105]. Viruses of the PR-2B clade of DENV serotype 2 produce subgenomic flavivirus RNA (sfRNA) that binds to tripartite motif-containing protein 25 (TRIM25) and prevents its deubiquitination [80]. Since TRIM25 is repressed while ubiquitinated [106], sfRNA expression maintains TRIM25 repression, impairing optimal activation of RIG-I and IFN production. TRIM25 regulates other processes such as NF-κB signaling [107] and cancer cell growth [108], so it would be interesting to see if TRIM25 antagonism affects its role in other pathways that benefit viral replication. The sfRNA of two other flaviviruses, West Nile virus (WNV) and Zika virus (ZIKV), have also been determined to negatively regulate the interferon response [109,110]. However, TRIM25 was either not investigated or did not bind in these studies, leaving the possibility of other pathways being targeted by sfRNA. 

### 4.2. NS1

The cytosolic DNA-detecting molecule, cyclic GMP-AMP synthase (cGAS), has been shown to inhibit various RNA viruses including flaviviruses [111]. ZIKV NS1 mediates the derepression of the inflammasome protease component, caspase-1, that has been shown to cleave cGAS [77]. Caspase-1 is silenced in an unstimulated state by K11-linked polyubiquitination that marks it for degradation by the proteasome [77]. Recruitment of ubiquitin-specific protease 8 (USP8) by ZIKV NS1 during infection leads to removal of K11-linked ubiquitin, and this correlates with increased cGAS cleavage. Furthermore, caspase-1 knockout leads to greater expression of IFN-β during ZIKV infection. Neither DENV infection nor ectopic expression of DENV NS1 have an effect on caspase-1 abundance, suggesting that this mechanism may be ZIKV specific. 

WNV also utilizes the NS1 protein to antagonize PRR recognition through a ubiquitin-mediated mechanism [82]. NS1 interacts with MDA5 and RIG-I in the cytoplasm and inhibits their expression. NS1 promotes the degradation of RIG-I, but not through K48-linked ubiquitination. Instead, there is a global decrease in K63-linked ubiquitination of RIG-I that appears linked to its degradation. This degradation is ameliorated with proteasome inhibitor treatment, supporting proteasome involvement. However, the results are less clear for MDA5, as proteasome inhibitor treatment does not increase MDA5 protein levels, suggesting that another degradative path such as the autophagy-lysosomal pathway may be involved.

### 4.3. NS2B, NS3 and NS2B3

Downstream adaptor proteins of the IFN production pathway are also targeted by flaviviruses through ubiquitin-mediated mechanisms. The viral NS2B3 complex is composed of the protease NS3 and the cofactor NS2B, and their interaction is essential for the serine protease catalytic activity [78]. Direct K27-linked polyubiquitination of DENV NS3 enhances recruitment of NS2B to form the viral-encoded protease NS2B3 [112]. The NS2B3 complexes of ZIKV and DENV are known to mediate cleavage of human STING [9,113,114]. Furthermore, K27-linked polyubiquitination of NS3 leads to increased STING binding and possibly its cleavage [112].

ZIKV was also shown to target STING and MAVS for degradation through the NS2B3 complex in a recent study [79]. However, the NS2B3 complex is not necessary for both interactions. NS3 interacts with MAVS, while NS2B3 interacts with STING. Both host-viral protein interactions lead to K48-linked ubiquitination and proteasome-mediated degradation of the host proteins. Another consequence of the NS2B-STING interaction is that other MAVS and STING ubiquitin linkages are affected. For MAVS, its K11-linked ubiquitination decreases, while its K29-linked polyubiquitination increases. For STING, K63- and K29-linked polyubiquitination both decrease. Since downstream signal transduction by STING also requires K63-linked ubiquitination [115], NS2B3 inhibits both the activation and abundance of STING.

As an additional example of protease-dependent antagonism, the flavivirus-related hepatitis C virus (HCV) can abrogate K63-linked polyubiquitination of RIG-I and downstream signaling. It does this through cleavage of Riplet by the HCV serine protease NS3-NS4A complex that resembles that of flavivirus NS2B3 [83]. While NS3-NS4A has not been demonstrated to cleave STING like NS2B3, it does cleave MAVS [116] indicating that this may be a shared antagonism mechanism within the Flaviviridae family. In a more recent study, the NS4A transmembrane domain of NS3-NS4A was determined to be critical for Riplet antagonism but evidence also suggests that Riplet may be involved in a pathway separate from RIG-I/MAVS but that still dependent on TBK1 and IRF3 [84]. Since TBK1 requires ubiquitination for its activation [117] it is possible that Riplet contributes to this directly, but how it would mediate this without the PAMP-recognition function of RIG-I is still a mystery. Ubiquitin-independent functions of Riplet have been determined [89] and it may be mediating these effects on TBK1 and IRF3 through an analogous mechanism.

### 4.4. NS5

DENV NS5 mediates degradation of the spliceosome protein RNA-binding motif 10 (RBM10) [81]. RBM10 has an established negative regulatory role in polyamine synthesis, an effect which can impact viral replication [81,118]. While RBM10 was not previously implicated in RIG-I signaling, the researchers found that the mRNA expression of IFN-I, RIG-I, and the NF-κB-activated gene, interleukin 8, were correlated with RBM10 expression during DENV infection or poly(I:C) treatment. RBM10 was also found to bind RIG-I directly, and this association was correlated with RBM10 concentration-dependent RIG-I ubiquitination. Such results suggest that RBM10 plays a role in activating RIG-I in addition to negative regulation of polyamine synthesis, and that DENV NS5 counters these antiviral actions by mediating ubiquitin-proteasomal degradation of RBM10. 

## 5. Ubiquitin-Mediated Antagonism of IFN-I Signaling by Flaviviruses

Flaviviruses use ubiquitin to evade IFN-I signaling in addition to IFN-I production. STAT proteins are often the major targets for degradation through different mechanisms. However, STAT antagonism is not restricted to degradation. There are other IFN-I signal pathway proteins as well as auxiliary proteins that flaviviruses target. Together, many of these mechanisms are host specific, highlighting the importance of the ubiquitin system in flavivirus host tropism (Figure 3).

### 5.1. NS5

When DENV NS5 is expressed alone, it is able to bind human STAT2. However, it has to be cleaved away from a polyprotein for it to target STAT2 for proteasome-mediated degradation [13]. The host component that tied both NS5-STAT2 binding and the degradation of STAT2 together is ubiquitin protein ligase E3 component N-recognin 4 (UBR4), a potential E3 ubiquitin ligase of the N-recognin family [15]. While the UBR4-STAT2-NS5 complex is essential for STAT2 degradation, UBR4 contains no HECT nor a RING catalytic domain to perform ubiquitination unlike other verified E3 ligases of the N-recognin family that are involved in protein degradation [119]. DENV2 NS5-mediated STAT2 degradation is species specific, as NS5 binds and degrades human but not mouse STAT2 [16]. 

ZIKV NS5 also suppresses IFN-I signaling through STAT2 degradation. As with DENV NS5, ZIKV NS5 induces proteasomal degradation of human STAT2 but not mouse STAT2 [17,18]. However, UBR4 does not appear to be involved in the process, indicating that other host E3 or E2 proteins are involved. Furthermore, ZIKV NS5 does not require proteolytic processing to mediate the degradation of STAT2 as DENV NS5 does [16].

Another avenue used by flaviviruses, as well as the distantly related hepatitis C virus (HCV), to degrade STAT2 involves the upregulation of a negative regulator of interferon signaling. HCV is a member of the Flaviviridae family, and shares similarities in genome structure, virion morphology, and replication strategies to flaviviruses that may manifest in similar antagonism pathways. IFN-I treatment of hepatocytes infected with HCV led to degradation of STAT2 after its relocation to the nucleus [96] PDLIM2, an E3 ubiquitin ligase, is known to ubiquitinate STAT1 and STAT4 for targeted proteasomal degradation in mice [120,121] and NF-κB [122,123] in human cells. Since PDLIM2 interacts with STAT2 upon IFN-I treatment, that could explain the increased levels of polyubiquitinated, nuclear STAT2 that were seen following IFN-I or MG132 treatment, or during HCV infection. Infection with HCV, DENV, or ZIKA all showed a MOI-dependent upregulation of PDLIM2 mRNA levels. Importantly, these changes in PDLIM2 were not from ISG production, supporting that PDLIM2 might be targeted by these viruses to antagonize IFN signaling. The exact mechanism of how HCV or flaviviruses influence PDLIM2 upregulation is unknown, but the PDLIM2-associated benefit to infectivity for all three viruses indicate that this may be a conserved mechanism among Flaviviridae family members [96].

Targeting integrated post-translational modification regulatory pathways, for example phosphorylation and ubiquitination, allow viruses more flexibility to achieve the same or greater antagonistic effects against host IFN signaling. For example, heat shock protein 90 (HSP90), a protein responsible for JAK stabilization, is bound but not degraded by NS5 in the flaviviruses WNV, ZIKV, and Japanese encephilitis virus [97]. In the absence of HSP90 binding, JAKs are destabilized and degraded via the ubiquitin-proteasome system. Since JAKs relay IFN and other various cytokine signals to STATs through phosphorylation, the signal transduction is terminated in their absence. In one study, the researchers uncovered the ability of diverse flaviviruses to target HSP90 for inhibition of the JAK/STAT signaling pathways along with other pro-inflammatory and immune-regulating cytokine signaling pathways. These results demonstrate that ubiquitin-mediated antagonism can also affect a broad scale of immune responses simultaneously and that regulatory proteins other than E3 ligases or E2 conjugating enzymes can be manipulated to affect the abundance of key pathway proteins via the ubiquitin-proteasome. 

While the majority of ubiquitin-mediated IFN signaling antagonism mechanisms involve degradation of host proteins, specific flaviviruses have alternatively adapted STAT sequestration. In the case of yellow fever virus (YFV), NS5 blocks the IFN-triggered transcription of ISGs via STAT2 binding [19]. The interaction of NS5 and STAT2 is dependent on the K63-polyubiquitination of the NS5 N-terminal region mediated by the host E3 enzyme tripartite motif 23 (TRIM23), an effect which is dependent on IFN-I treatment. Additionally, IFN-I-induced phosphorylation of STAT1 is also essential for STAT2 binding. Again, an intersection between phosphorylation- and ubiquitin-regulated pathways is hijacked for a potent flavivirus antagonism mechanism to IFN-I signaling. The role of YFV NS5 was also investigated in mice [27]. The lack of human-specific ubiquitin modifications of YFV NS5 may be a cause of poor YFV replication in murine cells and in vivo, indicating that host-specific ubiquitin regulation contributes to flavivirus host tropism.

### 5.2. NS4A

The flavivirus tick-borne encephalitis virus (TBEV) employs a STAT sequestration strategy through its NS4A protein [98]. TBEV NS4A inhibits STAT1/2 phosphorylation and dimerization. Similar to YFV NS5, TBEV NS4A requires ubiquitination of a lysine residue to bind STAT1/2. However, TBEV NS4A inhibits STAT1/2 phosphorylation, and K27-linked ubiquitination is utilized instead of K63-linked. Interestingly, TBEV infection enhances K27-linked ubiquitination while IFN-I treatment interferes with this modification. NS5 of TBEV also inhibits the JAK/STAT pathway [25], but flaviviruses often encode multiple antagonists of one pathway that function in different ways. While it seems that NS4A may be a redundant STAT inhibitor, the time- and IFN-dependent effects on NS4A ubiquitination, and thus STAT inhibition, may have larger implications in the TBEV life cycle. 

## 6. Ubiquitin-Mediated Antagonism of JAK/STAT Signaling in Insects

In mosquitos, ubiquitin plays a role in flavivirus signaling antagonism similar to that found in mammals. The mosquito immune system differs from mammals, but certain elements like Toll and JAK-STAT signal transduction pathways are maintained through homologs [124,125,126]. During WNV infection of *Culex* mosquito cells, many ubiquitin proteasome system (UPS) genes are upregulated including *Culex* Cul4 (*Cx*Cul4), an ortholog Cullin4A and Cullin4B [26]. Cullin proteins in mammals are known to associate with a multitude of other proteins to form multisubunit E3 enzymes called Cullin-RING ubiquitin ligases that mediate the selectivity and ubiquitination of substrates. *Cx*Cul4 functions as a negative regulator of the JAK/STAT pathway by mediating STAT degradation via the UPS. Whether *Cx*Cul4 does this through ubiquitinating STAT is unclear, but individual expression of WNV NS1 and NS5 led to significant increases in *Cx*Cul4 mRNA abundance, indicating that both proteins might act through this antagonism pathway within WNV-infected mosquitoes. In contrast, WNV infection in human cells does not lead to STAT degradation. WNV instead inhibits STAT1 phosphorylation accumulation via a NS5-mediated mechanism [12]. No ubiquitin component is involved in this WNV mechanism during infection of human cells, but this contrast of host-specific approaches to STAT antagonism demonstrates that ubiquitin pathways are a part of many host post-translational modification regulatory systems that flaviviruses can hijack to optimize their antagonism strategies.

## 7. Conclusions

Diverse species of flaviviruses have a demonstrated dependence on the ubiquitin system for antagonism of the host immune response. While many flaviviruses use ubiquitin-mediated antagonism, the specific mechanism varies between flavivirus species. Furthermore, multiple effectors from the same viral species can manipulate ubiquitin during the infection of a single host. DENV is such an example, where NS5, NS2B3, and sfRNA each have different ubiquitin-mediated antagonism mechanisms [13,15,80,81,112]. Most of these antagonism mechanisms appear to be adapted independently by different flavivirus species and in a host-specific manner, although some conserved mechanisms may exist amongst flaviviruses and will require further investigation to validate [96,97]. It is well established that ubiquitin is important for the successful infection of flaviviruses, but investigating additional host-specific aspects could benefit efforts to combat flavivirus-mediated disease among the human population. First, elucidating essential host ubiquitin factors for flavivirus antagonism would provide targets for the development of host-directed antiviral therapeutics. Second, epidemiological surveying of flavivirus reservoirs for potential emergence of human-permissive strains could include assessing the ability of a strain to manipulate these ubiquitinating enzymes. In both cases, determining the host ubiquitin-regulating proteins and their molecular interactions with flavivirus effectors are necessary.

## Data Availability

Not applicable.

## References

[1] Gubler D.J. (2008). Dengue/Dengue Haemorrhagic Fever: History and Current Status. Novartis Found. Symp..

[2] Guarner J., Hale G.L. (2019). Four human diseases with significant public health impact caused by mosquito-borne flaviviruses: West Nile, Zika, dengue and yellow fever. Semin. Diagn. Pathol..

[3] Dub T., Ollgren J., Huusko S., Uusitalo R., Siljander M., Vapalahti O., Sane J. (2020). Game Animal Density, Climate, and Tick-Borne Encephalitis in Finland, 2007–2017. Emerg. Infect. Dis..

[4] Pierson T.C., Diamond M.S. (2020). The continued threat of emerging flaviviruses. Nat. Microbiol..

[5] Guzman M.G., Halstead S.B., Artsob H., Buchy P., Farrar J., Gubler D.J., Hunsperger E., Kroeger A., Margolis H.S., Martínez E. (2010). Dengue: A continuing global threat. Nat. Rev. Genet..

[6] MacKenzie J.S., Gubler D.J., Petersen L.R. (2004). Emerging flaviviruses: The spread and resurgence of Japanese encephalitis, West Nile and dengue viruses. Nat. Med..

[7] Lira-Vieira A.R., Gurgel-Gonçalves R., Moreira I.M., Yoshizawa M.A.C., Coutinho M.L., Prado P.S., De Souza J.L., Chaib A.J.D.M., Moreira J.S., De Castro C.N. (2013). Ecological aspects of mosquitoes (Diptera: Culicidae) in the gallery forest of Brasilia National Park, Brazil, with an emphasis on potential vectors of yellow fever. Rev. Soc. Bras. Med. Trop..

[8] Barrows N.J., Campos R.K., Liao K.-C., Prasanth K.R., Soto-Acosta R., Yeh S.-C., Schott-Lerner G., Pompon J., Sessions O.M., Bradrick S.S. (2018). Biochemistry and Molecular Biology of Flaviviruses. Chem. Rev..

[9] Aguirre S., Maestre A.M., Pagni S., Patel J.R., Savage T., Gutman D., Maringer K., Bernal-Rubio D., Shabman R.S., Simon V. (2012). DENV Inhibits Type I IFN Production in Infected Cells by Cleaving Human STING. PLoS Pathog..

[10] Yu C.-Y., Chang T.-H., Liang J.-J., Chiang R.-L., Lee Y.-L., Liao C.-L., Lin Y.-L. (2012). Dengue Virus Targets the Adaptor Protein MITA to Subvert Host Innate Immunity. PLoS Pathog..

[11] Lubick K.J., Robertson S.J., McNally K.L., Freedman B.A., Rasmussen A.L., Taylor R.T., Walts A.D., Tsuruda S., Sakai M., Ishizuka M. (2015). Flavivirus Antagonism of Type I Interferon Signaling Reveals Prolidase as a Regulator of IFNAR1 Surface Expression. Cell Host Microbe.

[12] Laurent-Rolle M., Boer E.F., Lubick K.J., Wolfinbarger J.B., Carmody A.B., Rockx B., Liu W., Ashour J., Shupert W.L., Holbrook M.R. (2010). The NS5 Protein of the Virulent West Nile Virus NY99 Strain Is a Potent Antagonist of Type I Interferon-Mediated JAK-STAT Signaling. J. Virol..

[13] Ashour J., Laurent-Rolle M., Shi P.-Y., Garcia-Sastre A. (2009). NS5 of Dengue Virus Mediates STAT2 Binding and Degradation. J. Virol..

[14] Mazzon M., Jones M., Davidson A., Chain B., Jacobs M. (2009). Dengue Virus NS5 Inhibits Interferon-α Signaling by Blocking Signal Transducer and Activator of Transcription 2 Phosphorylation. J. Infect. Dis..

[15] Morrison J., Laurent-Rolle M., Maestre A.M., Rajsbaum R., Pisanelli G., Simon V., Mulder L.C.F., Fernandez-Sesma A., García-Sastre A. (2013). Dengue Virus Co-opts UBR4 to Degrade STAT2 and Antagonize Type I Interferon Signaling. PLoS Pathog..

[16] Ashour J., Morrison J., Laurent-Rolle M., Belicha-Villanueva A., Plumlee C.R., Bernal-Rubio D., Williams K.L., Harris E., Fernandez-Sesma A., Schindler C. (2010). Mouse STAT2 Restricts Early Dengue Virus Replication. Cell Host Microbe.

[17] Grant A., Ponia S.S., Tripathi S., Balasubramaniam V., Miorin L., Sourisseau M., Schwarz M.C., Sánchez-Seco M.P., Evans M.J., Best S.M. (2016). Zika Virus Targets Human STAT2 to Inhibit Type I Interferon Signaling. Cell Host Microbe.

[18] Kumar A., Hou S., Airo A.M., Limonta D., Mancinelli V., Branton W., Power C., Hobman T.C. (2016). Zika virus inhibits type-I interferon production and downstream signaling. EMBO Rep..

[19] Laurent-Rolle M., Morrison J., Rajsbaum R., MacLeod J.M.L., Pisanelli G., Pham A., Ayllon J., Miorin L., Martínez-Romero C., Tenoever B.R. (2014). The Interferon Signaling Antagonist Function of Yellow Fever Virus NS5 Protein Is Activated by Type I Interferon. Cell Host Microbe.

[20] Lin R.-J., Chang B.-L., Yu H.-P., Liao C.-L., Lin Y.-L. (2006). Blocking of Interferon-Induced Jak-Stat Signaling by Japanese Encephalitis Virus NS5 through a Protein Tyrosine Phosphatase-Mediated Mechanism. J. Virol..

[21] Yang T.-C., Li S.-W., Lai C.-C., Lu K.-Z., Chiu M.-T., Hsieh T.-H., Wan L., Lin C.-W. (2013). Proteomic analysis for Type I interferon antagonism of Japanese encephalitis virus NS5 protein. Proteomics.

[22] Best S.M., Morris K.L., Shannon J.G., Robertson S.J., Mitzel D.N., Park G.S., Boer E., Wolfinbarger J.B., Bloom M.E. (2005). Inhibition of Interferon-Stimulated JAK-STAT Signaling by a Tick-Borne Flavivirus and Identification of NS5 as an Interferon Antagonist. J. Virol..

[23] Park G.S., Morris K.L., Hallett R.G., Bloom M.E., Best S.M. (2007). Identification of Residues Critical for the Interferon Antagonist Function of Langat Virus NS5 Reveals a Role for the RNA-Dependent RNA Polymerase Domain. J. Virol..

[24] Valmas C., Grosch M.N., Schümann M., Olejnik J., Martinez O., Best S.M., Krähling V., Basler C.F., Mühlberger E. (2010). Marburg Virus Evades Interferon Responses by a Mechanism Distinct from Ebola Virus. PLoS Pathog..

[25] Werme K., Wigerius M., Johansson M. (2008). Tick-borne encephalitis virus NS5 associates with membrane protein scribble and impairs interferon-stimulated JAK-STAT signalling. Cell. Microbiol..

[26] Paradkar P.N., Duchemin J.-B., Rodriguez-Andres J., Trinidad L., Walker P.J. (2015). Cullin4 Is Pro-Viral during West Nile Virus Infection of Culex Mosquitoes. PLoS Pathog..

[27] Miorin L., Laurent-Rolle M., Pisanelli G., Co P.H., Albrecht R.A., García-Sastre A., Morrison J. (2019). Host-Specific NS5 Ubiquitination Determines Yellow Fever Virus Tropism. J. Virol..

[28] Chaudhary V., Yuen K.-S., Chan J.F.-W., Chan C.-P., Wang P.-H., Cai J.-P., Zhang S., Liang M., Kok K.-H., Yuen K.-Y. (2017). Selective Activation of Type II Interferon Signaling by Zika Virus NS5 Protein. J. Virol..

[29] Ye J., Chen Z., Li Y., Zhao Z., He W., Zohaib A., Cao S. (2017). Japanese Encephalitis Virus NS5 Inhibits Type I Interferon (IFN) Production by Blocking the Nuclear Translocation of IFN Regulatory Factor 3 and NF-kappaB. J. Virol..

[30] Finley D., Ciechanover A., Varshavsky A. (1984). Thermolability of ubiquitin-activating enzyme from the mammalian cell cycle mutant ts85. Cell.

[31] Hershko A., Leshinsky E., Ganoth D., Heller H. (1984). ATP-dependent degradation of ubiquitin-protein conjugates. Proc. Natl. Acad. Sci. USA.

[32] Varshavsky A. (2006). The early history of the ubiquitin field. Protein Sci..

[33] Akutsu M., Dikic I., Bremm A. (2016). Ubiquitin chain diversity at a glance. J. Cell Sci..

[34] Cagno V., Tseligka E.D., Bettex Q., Huang S., Constant S., Tapparel C. (2019). Growth of Zika virus in human reconstituted respiratory, intestinal, vaginal and neural tissues. Clin. Microbiol. Infect..

[35] Gustin J.K.P., Moses A.V.P., Früh K.P., Douglas J.L.P. (2011). Viral Takeover of the Host Ubiquitin System. Front. Microbiol..

[36] McGinty R.K., Henrici R.C., Tan S. (2014). Crystal structure of the PRC1 ubiquitylation module bound to the nucleosome. Nat. Cell Biol..

[37] Stewart M.D., Ritterhoff T., Klevit R.E., Brzovic P.S. (2016). E2 enzymes: More than just middle men. Cell Res..

[38] Ebner P., Versteeg G.A., Ikeda F. (2017). Ubiquitin enzymes in the regulation of immune responses. Crit. Rev. Biochem. Mol. Biol..

[39] Locke M., Toth J.I., Petroski M.D. (2014). Lys11- and Lys48-linked ubiquitin chains interact with p97 during endoplasmic-reticulum-associated degradation. Biochem. J..

[40] Zhao C., Jia M., Song H., Yu Z., Wang W., Li Q., Zhang L., Zhao W., Cao X. (2017). The E3 Ubiquitin Ligase TRIM40 Attenuates Antiviral Immune Responses by Targeting MDA5 and RIG-I. Cell Rep..

[41] Matsumoto M.L., Wickliffe K.E., Dong K.C., Yu C., Bosanac I., Bustos D., Phu L., Kirkpatrick D.S., Hymowitz S.G., Rape M. (2010). K11-Linked Polyubiquitination in Cell Cycle Control Revealed by a K11 Linkage-Specific Antibody. Mol. Cell.

[42] Kravtsova-Ivantsiv Y., Ciechanover A. (2012). Non-canonical ubiquitin-based signals for proteasomal degradation. J. Cell Sci..

[43] Grumati P., Dikic I. (2018). Ubiquitin signaling and autophagy. J. Biol. Chem..

[44] Van Huizen M., Kikkert M. (2019). The Role of Atypical Ubiquitin Chains in the Regulation of the Antiviral Innate Immune Response. Front. Cell Dev. Biol..

[45] Qin Y., Zhou M.-T., Hu M.-M., Hu Y.-H., Zhang J., Guo L., Zhong B., Shu H.-B. (2014). RNF26 Temporally Regulates Virus-Triggered Type I Interferon Induction by Two Distinct Mechanisms. PLoS Pathog..

[46] Jin S., Tian S., Chen Y., Zhang C., Xie W., Xia X., Cui J., Wang R. (2016). USP 19 modulates autophagy and antiviral immune responses by deubiquitinating Beclin-1. EMBO J..

[47] Kensche T., Tokunaga F., Ikeda F., Goto E., Iwai K., Dikic I. (2012). Analysis of nuclear factor-kappaB (NF-kappaB) essential modulator (NEMO) binding to linear and lysine-linked ubiquitin chains and its role in the activation of NF-kappaB. J. Biol. Chem..

[48] Belgnaoui S.M., Paz S., Samuel S., Goulet M.-L., Sun Q., Kikkert M., Iwai K., Dikic I., Hiscott J., Lin R. (2012). Linear Ubiquitination of NEMO Negatively Regulates the Interferon Antiviral Response through Disruption of the MAVS-TRAF3 Complex. Cell Host Microbe.

[49] Liu S., Jiang M., Wang W., Liu W., Song X., Ma Z., Zhang S., Liu L., Liu Y., Cao X. (2018). Nuclear RNF2 inhibits interferon function by promoting K33-linked STAT1 disassociation from DNA. Nat. Immunol..

[50] Lin M., Zhao Z., Yang Z., Meng Q., Tan P., Xie W., Qin Y., Wang R.-F., Cui J. (2016). USP38 Inhibits Type I Interferon Signaling by Editing TBK1 Ubiquitination through NLRP4 Signalosome. Mol. Cell.

[51] Lei C.Q., Wu X., Zhong X., Jiang L., Zhong B., Shu H.B. (2019). USP19 Inhibits TNF-alpha- and IL-1beta-Triggered NF-kappaB Activation by Deubiquitinating TAK1. J. Immunol..

[52] Chen Y., Wang L., Jin J., Luan Y., Chen C., Li Y., Chu H., Wang X., Liao G., Yu Y. (2017). p38 inhibition provides anti–DNA virus immunity by regulation of USP21 phosphorylation and STING activation. J. Exp. Med..

[53] Sun H., Zhang Q., Jing Y.-Y., Zhang M., Wang H.-Y., Cai Z., Liuyu T., Zhang Z.-D., Xiong T.-C., Wu Y. (2017). USP13 negatively regulates antiviral responses by deubiquitinating STING. Nat. Commun..

[54] Liu J., Han C., Xie B., Wu Y., Liu S., Chen K., Xia M., Zhuang Y., Song L., Li Z. (2014). Rhbdd3 controls autoimmunity by suppressing the production of IL-6 by dendritic cells via K27-linked ubiquitination of the regulator NEMO. Nat. Immunol..

[55] Arimoto K.-I., Funami K., Saeki Y., Tanaka K., Okawa K., Takeuchi O., Akira S., Murakami Y., Shimotohno K. (2010). Polyubiquitin conjugation to NEMO by triparite motif protein 23 (TRIM23) is critical in antiviral defense. Proc. Natl. Acad. Sci. USA.

[56] Wang Q., Huang L., Hong Z., Lv Z., Mao Z., Tang Y., Zhou Q. (2017). The E3 ubiquitin ligase RNF185 facilitates the cGAS-mediated innate immune response. PLoS Pathog..

[57] Xue B., Li H., Guo M., Wang J., Xu Y., Zou X., Deng R., Li G., Zhu H. (2018). TRIM21 Promotes Innate Immune Response to RNA Viral Infection through Lys27-Linked Polyubiquitination of MAVS. J. Virol..

[58] Hwang S.Y., Hertzog P.J., Holland K.A., Sumarsono S.H., Tymms M.J., Hamilton J.A., Whitty G., Bertoncello I., Kola I. (1995). A null mutation in the gene encoding a type I interferon receptor component eliminates antiproliferative and antiviral responses to interferons alpha and beta and alters macrophage responses. Proc. Natl. Acad. Sci. USA.

[59] Muller U., Steinhoff U., Reis L., Hemmi S., Pavlovic J., Zinkernagel R., Aguet M. (1994). Functional role of type I and type II interferons in antiviral defense. Science.

[60] Huang S., Hendriks W., Althage A., Hemmi S., Bluethmann H., Kamijo R., Vilcek J., Zinkernagel R., Aguet M. (1993). Immune response in mice that lack the interferon-gamma receptor. Science.

[61] Isaacs A., Lindenmann J. (1957). Virus interference. I. The interferon. Proc. R. Soc. Lond. Ser. B Boil. Sci..

[62] Dalton D., Pitts-Meek S., Keshav S., Figari I., Bradley A., Stewart T. (1993). Multiple defects of immune cell function in mice with disrupted interferon-gamma genes. Science.

[63] Coccia E.M., Severa M., Giacomini E., Monneron D., Remoli M.E., Julkunen I., Uzé G. (2004). Viral infection and Toll-like receptor agonists induce a differential expression of type I and lambda interferons in human plasmacytoid and monocyte-derived dendritic cells. Eur. J. Immunol..

[64] Borden E.C., Sen G.C., Uze G., Silverman R.H., Ransohoff R.M., Foster G.R., Stark G.R. (2007). Interferons at age 50: Past, current and future impact on biomedicine. Nat. Rev. Drug Discov..

[65] Kalliolias G.D., Ivashkiv L.B. (2010). Overview of the biology of type I interferons. Arthritis Res. Ther..

[66] Bekisz J., Schmeisser H., Hernandez J., Goldman N.D., Zoon K.C. (2004). Mini ReviewHuman Interferons Alpha, Beta and Omega. Growth Factors.

[67] Yoneyama M., Kikuchi M., Natsukawa T., Shinobu N., Imaizumi T., Miyagishi M., Taira K., Akira S., Fujita T. (2004). The RNA helicase RIG-I has an essential function in double-stranded RNA-induced innate antiviral responses. Nat. Immunol..

[68] Pichlmair A., Schulz O., Tan C.P., Näslund T.I., Liljeström P., Weber F., E Sousa C.R. (2006). RIG-I-Mediated Antiviral Responses to Single-Stranded RNA Bearing 5’-Phosphates. Science.

[69] Kato H., Takeuchi O., Sato S., Yoneyama M., Yamamoto M., Matsui K., Uematsu S., Jung A., Kawai T., Ishii K.J. (2006). Differential roles of MDA5 and RIG-I helicases in the recognition of RNA viruses. Nat. Cell Biol..

[70] Sun X., Xian H., Tian S., Sun T., Qin Y., Zhang S., Cui J. (2016). A Hierarchical Mechanism of RIG-I Ubiquitination Provides Sensitivity, Robustness and Synergy in Antiviral Immune Responses. Sci. Rep..

[71] Liu Y., Olagnier D., Lin R. (2017). Host and Viral Modulation of RIG-I-Mediated Antiviral Immunity. Front. Immunol..

[72] Gack M.U., Shin Y.C., Joo C.-H., Urano T., Liang C., Sun L., Takeuchi O., Akira S., Chen Z., Inoue S. (2007). TRIM25 RING-finger E3 ubiquitin ligase is essential for RIG-I-mediated antiviral activity. Nat. Cell Biol..

[73] Gack M.U., Kirchhofer A., Shin Y.C., Inn K.-S., Liang C., Cui S., Myong S., Ha T., Hopfner K.-P., Jung J.U. (2008). Roles of RIG-I N-terminal tandem CARD and splice variant in TRIM25-mediated antiviral signal transduction. Proc. Natl. Acad. Sci. USA.

[74] Xian H., Xie W., Yang S., Liu Q., Xia X., Jin S., Sun T., Cui J. (2017). Stratified ubiquitination of RIG-I creates robust immune response and induces selective gene expression. Sci. Adv..

[75] Meylan E., Curran J., Hofmann K., Moradpour D., Binder M., Bartenschlager R., Tschopp J. (2005). Cardif is an adaptor protein in the RIG-I antiviral pathway and is targeted by hepatitis C virus. Nat. Cell Biol..

[76] Seth R.B., Sun L., Ea C.K., Chen Z.J. (2005). Identification and characterization of MAVS, a mitochondrial antiviral signaling protein that activates NF-kappaB and IRF 3. Cell.

[77] Zheng Y., Liu Q., Wu Y., Ma L., Zhang Z., Liu T., Jin S., She Y., Li Y., Cui J. (2018). Zika virus elicits inflammation to evade antiviral response by cleaving cGAS via NS 1-caspase-1 axis. EMBO J..

[78] Erbel P., Schiering N., D’Arcy A., Renatus M., Kroemer M., Lim S.P., Yin Z., Keller T.H., Vasudevan S.G., Hommel U. (2006). Structural basis for the activation of flaviviral NS3 proteases from dengue and West Nile virus. Nat. Struct. Mol. Biol..

[79] Li W., Li N., Dai S., Hou G., Guo K., Chen X., Yi C., Liu W., Deng F., Wu Y. (2019). Zika virus circumvents host innate immunity by targeting the adaptor proteins MAVS and MITA. FASEB J..

[80] Manokaran G., Finol E., Wang C., Gunaratne J., Bahl J., Ong E.Z., Tan H.C., Sessions O.M., Ward A.M., Gubler D.J. (2015). Dengue subgenomic RNA binds TRIM25 to inhibit interferon expression for epidemiological fitness. Science.

[81] Pozzi B., Bragado L., Mammi P., Torti M.F., Gaioli N., Gebhard L.G., Solá M.E.G., Vaz-Drago R., Iglesias N.G., García C.C. (2020). Dengue virus targets RBM10 deregulating host cell splicing and innate immune response. Nucleic Acids Res..

[82] Zhang H.-L., Ye H.-Q., Liu S.-Q., Deng C.-L., Li X.-D., Shi P.-Y., Zhang B. (2017). West Nile Virus NS1 Antagonizes Interferon Beta Production by Targeting RIG-I and MDA5. J. Virol..

[83] Oshiumi H., Miyashita M., Matsumoto M., Seya T. (2013). A Distinct Role of Riplet-Mediated K63-Linked Polyubiquitination of the RIG-I Repressor Domain in Human Antiviral Innate Immune Responses. PLoS Pathog..

[84] Vazquez C., Tan C.Y., Horner S.M. (2019). Hepatitis C Virus Infection Is Inhibited by a Noncanonical Antiviral Signaling Pathway Targeted by NS3-NS4A. J. Virol..

[85] Oshiumi H., Matsumoto M., Hatakeyama S., Seya T. (2009). Riplet/RNF135, a RING finger protein, ubiquitinates RIG-I to promote interferon-beta induction during the early phase of viral infection. J. Biol. Chem..

[86] Gao D., Yang Y.K., Wang R.P., Zhou X., Diao F.C., Li M.D., Chen D.Y. (2009). REUL is a novel E3 ubiquitin ligase and stimulator of retinoic-acid-inducible gene-I. PLoS ONE.

[87] Oshiumi H., Miyashita M., Inoue N., Okabe M., Matsumoto M., Seya T. (2010). The Ubiquitin Ligase Riplet Is Essential for RIG-I-Dependent Innate Immune Responses to RNA Virus Infection. Cell Host Microbe.

[88] Hayman T.J., Hsu A.C., Kolesnik T.B., Dagley L.F., Willemsen J., Tate M.D., Baker P.J., Kershaw N.J., Kedzierski L., I Webb A. (2019). RIPLET, and not TRIM25, is required for endogenous RIG-I-dependent antiviral responses. Immunol. Cell Biol..

[89] Cadena C., Ahmad S., Xavier A., Willemsen J., Park S., Park J.W., Oh S.-W., Fujita T., Hou F., Binder M. (2019). Ubiquitin-Dependent and -Independent Roles of E3 Ligase RIPLET in Innate Immunity. Cell.

[90] Vazquez C., Horner S.M. (2015). MAVS Coordination of Antiviral Innate Immunity. J. Virol..

[91] Hiscott J. (2007). Triggering the Innate Antiviral Response through IRF-3 Activation. J. Biol. Chem..

[92] Zhong B., Yang Y., Li S., Wang Y.-Y., Li Y., Diao F., Lei C., He X., Zhang L., Tien P. (2008). The Adaptor Protein MITA Links Virus-Sensing Receptors to IRF3 Transcription Factor Activation. Immunity.

[93] Sun W., Li Y., Chen L., Chen H., You F., Zhou X., Zhou Y., Zhai Z., Chen D., Jiang Z. (2009). ERIS, an endoplasmic reticulum IFN stimulator, activates innate immune signaling through dimerization. Proc. Natl. Acad. Sci. USA.

[94] Lai J., Wang M., Huang C., Wu C., Hung L., Yang C., Ke P., Luo S., Liu S., Ho L. (2018). Infection with the dengue RNA virus activates TLR9 signaling in human dendritic cells. EMBO Rep..

[95] Sun B., Sundström K.B., Chew J.J., Bist P., Gan E.S., Tan H.C., Goh K.C., Chawla T., Tang C.K., Ooi E.E. (2017). Dengue virus activates cGAS through the release of mitochondrial DNA. Sci. Rep..

[96] Joyce M.A., Berry-Wynne K.M., Dos Santos T., Addison W.R., McFarlane N., Hobman T., Tyrrell D.L. (2019). HCV and flaviviruses hijack cellular mechanisms for nuclear STAT2 degradation: Up-regulation of PDLIM2 suppresses the innate immune response. PLoS Pathog..

[97] Roby J.A., Esser-Nobis K., Dewey-Verstelle E.C., Fairgrieve M.R., Schwerk J., Lu A.Y., Soveg F.W., Hemann E.A., Hatfield L.D., Keller B.C. (2020). Flavivirus Nonstructural Protein NS5 Dysregulates HSP90 to Broadly Inhibit JAK/STAT Signaling. Cells.

[98] Yang Q., You J., Zhou Y., Wang Y., Pei R., Chen X., Chen J. (2020). Tick-borne encephalitis virus NS4A ubiquitination antagonizes type I interferon-stimulated STAT1/2 signalling pathway. Emerg. Microbes. Infect..

[99] Becquart P., Wauquier N., Nkoghe D., Ndjoyi-Mbiguino A., Padilla C., Souris M., Leroy E.M. (2010). Acute dengue virus 2 infection in Gabonese patients is associated with an early innate immune response, including strong interferon alpha production. BMC Infect. Dis..

[100] Lobigs M., Müllbacher A., Wang Y., Pavy M., Lee E. (2003). Role of type I and type II interferon responses in recovery from infection with an encephalitic flavivirus. J. Gen. Virol..

[101] Samuel M.A., Diamond M.S. (2005). Alpha/Beta Interferon Protects against Lethal West Nile Virus Infection by Restricting Cellular Tropism and Enhancing Neuronal Survival. J. Virol..

[102] Shresta S., Kyle J.L., Snider H.M., Basavapatna M., Beatty P.R., Harris E. (2004). Interferon-Dependent Immunity Is Essential for Resistance to Primary Dengue Virus Infection in Mice, Whereas T- and B-Cell-Dependent Immunity Are Less Critical. J. Virol..

[103] Gack M.U., Albrecht R.A., Urano T., Inn K.-S., Huang I.-C., Carnero E., Farzan M., Inoue S., Jung J.U., García-Sastre A. (2009). Influenza A Virus NS1 Targets the Ubiquitin Ligase TRIM25 to Evade Recognition by the Host Viral RNA Sensor RIG-I. Cell Host Microbe.

[104] Inn K.S., Lee S.H., Rathbun J.Y., Wong L.Y., Toth Z., Machida K., Jung J.U. (2011). Inhibition of RIG-I-mediated signaling by Kaposi’s sarcoma-associated herpesvirus-encoded deubiquitinase ORF64. J. Virol..

[105] Sun L., Xing Y., Chen X., Zheng Y., Yang Y., Nichols D.B., Clementz M.A., Banach B.S., Li K., Baker S.C. (2012). Coronavirus Papain-like Proteases Negatively Regulate Antiviral Innate Immune Response through Disruption of STING-Mediated Signaling. PLoS ONE.

[106] Pauli E.-K., Chan Y.K., Davis M.E., Gableske S., Wang M.K., Feister K.F., Gack M.U. (2014). The Ubiquitin-Specific Protease USP15 Promotes RIG-I-Mediated Antiviral Signaling by Deubiquitylating TRIM25. Sci. Signal..

[107] Liu Y., Liu K., Huang Y., Sun M., Tian Q., Zhang S., Qin Y. (2020). TRIM25 Promotes TNF-alpha-Induced NF-kappaB Activation through Potentiating the K63-Linked Ubiquitination of TRAF2. J. Immunol..

[108] Liu Y., Tao S., Liao L., Li Y., Li H., Li Z., Lin L., Wan X., Yang X., Chen L. (2020). TRIM25 promotes the cell survival and growth of hepatocellular carcinoma through targeting Keap1-Nrf2 pathway. Nat. Commun..

[109] Schuessler A., Funk A., LaZear H.M., Cooper D.A., Torres S., Daffis S., Jha B.K., Kumagai Y., Takeuchi O., Hertzog P. (2012). West Nile Virus Noncoding Subgenomic RNA Contributes to Viral Evasion of the Type I Interferon-Mediated Antiviral Response. J. Virol..

[110] Donald C.L., Brennan B., Cumberworth S.L., Rezelj V.V., Clark J.J., Cordeiro M.T., França R.F.D.O., Pena L.J., Wilkie G.S., Filipe A.D.S. (2016). Full Genome Sequence and sfRNA Interferon Antagonist Activity of Zika Virus from Recife, Brazil. PLoS Neglected Trop. Dis..

[111] Schoggins J.W., MacDuff D.A., Imanaka N., Gainey M.D., Shrestha B., Eitson J.L., Mar K.B., Richardson R.B., Ratushny A.V., Litvak V. (2014). Pan-viral specificity of IFN-induced genes reveals new roles for cGAS in innate immunity. Nat. Cell Biol..

[112] Liu H., Zhang L., Sun J., Chen W., Li S., Wang Q., Yu H., Xia Z., Jin X., Wang C. (2017). Endoplasmic Reticulum Protein SCAP Inhibits Dengue Virus NS2B3 Protease by Suppressing Its K27-Linked Polyubiquitylation. J. Virol..

[113] Ding Q., Gaska J.M., Douam F., Wei L., Kim D., Balev M., Heller B., Ploss A. (2018). Species-specific disruption of STING-dependent antiviral cellular defenses by the Zika virus NS2B3 protease. Proc. Natl. Acad. Sci. USA.

[114] Stabell A.C., Meyerson N.R., Gullberg R.C., Gilchrist A.R., Webb K.J., Old W.M., Perera R., Sawyer S.L. (2018). Dengue viruses cleave STING in humans but not in nonhuman primates, their presumed natural reservoir. eLife.

[115] Zhang J., Hu M.-M., Wang Y.-Y., Shu H.-B. (2012). TRIM32 Protein Modulates Type I Interferon Induction and Cellular Antiviral Response by Targeting MITA/STING Protein for K63-linked Ubiquitination*. J. Biol. Chem..

[116] Li X.D., Sun L., Seth R.B., Pineda G., Chen Z.J. (2005). Hepatitis C virus protease NS3/4A cleaves mitochondrial antiviral signaling protein off the mitochondria to evade innate immunity. Proc. Natl. Acad. Sci. USA.

[117] Tu D., Zhu Z., Zhou A.Y., Yun C.-H., Lee K.-E., Toms A.V., Li Y., Dunn G.P., Chan E., Thai T. (2013). Structure and Ubiquitination-Dependent Activation of TANK-Binding Kinase 1. Cell Rep..

[118] Smirnova O.A., Keinanen T.A., Ivanova O.N., Hyvonen M.T., Khomutov A.R., Kochetkov S.N., Bartosch B., Ivanov A.V. (2017). Hepatitis C virus alters metabolism of biogenic polyamines by affecting expression of key enzymes of their metabolism. Biochem. Biophys. Res. Commun..

[119] Tasaki T., Mulder L.C.F., Iwamatsu A., Lee M.J., Davydov I.V., Varshavsky A., Muesing M., Kwon Y.T. (2005). A Family of Mammalian E3 Ubiquitin Ligases That Contain the UBR Box Motif and Recognize N-Degrons. Mol. Cell. Biol..

[120] Tanaka T., Soriano M.A., Grusby M.J. (2005). SLIM Is a Nuclear Ubiquitin E3 Ligase that Negatively Regulates STAT Signaling. Immunity.

[121] Guo H., Mi Z., Bowles D.E., Bhattacharya S.D., Kuo P.C. (2010). Osteopontin and Protein Kinase C Regulate PDLIM2 Activation and STAT1 Ubiquitination in LPS-treated Murine Macrophages. J. Biol. Chem..

[122] Tanaka T., Grusby M.J., Kaisho T. (2007). PDLIM2-mediated termination of transcription factor NF-kappaB activation by intranuclear sequestration and degradation of the p65 subunit. Nat. Immunol..

[123] Healy N.C., O’Connor R. (2009). Sequestration of PDLIM2 in the cytoplasm of monocytic/macrophage cells is associated with adhesion and increased nuclear activity of NF-kappaB. J. Leukoc. Biol..

[124] Fragkoudis R., Attarzadeh-Yazdi G., Nash A.A., Fazakerley J.K., Kohl A. (2009). Advances in dissecting mosquito innate immune responses to arbovirus infection. J. Gen. Virol..

[125] Paradkar P.N., Trinidad L., Voysey R., Duchemin J.-B., Walker P.J. (2012). Secreted Vago restricts West Nile virus infection in Culex mosquito cells by activating the Jak-STAT pathway. Proc. Natl. Acad. Sci. USA.

[126] Souza-Neto J.A., Sim S., Dimopoulos G. (2009). An evolutionary conserved function of the JAK-STAT pathway in anti-dengue defense. Proc. Natl. Acad. Sci. USA.

